# COVID-19 burnout, resilience, and psychological distress among Chinese college students

**DOI:** 10.3389/fpubh.2022.1009027

**Published:** 2022-11-16

**Authors:** YueYi Sun, ShuYue Zhu, GanXin ChenHuang, LiYa Zhu, ShuHan Yang, XiaoCong Zhang, Zheng Zheng

**Affiliations:** Department of Psychology, School of Medicine & Holistic Integrative Medicine, Nanjing University of Chinese Medicine, Nanjing, China

**Keywords:** COVID-19 burnout, resilience, mental health, students, China

## Abstract

**Background:**

Since the outbreak of coronavirus disease 2019 (COVID-19), Chinese college students have spent 3 years dealing with infection prevention. Some students have undergone quarantine due to the detection of new variants of COVID-19 and the rise in cases. This study examines pandemic-related isolation and its psychological impact on Chinese college students and explores the relationships among COVID-19 burnout, resilience, and psychological distress in Chinese college students during the pandemic.

**Methods:**

The COVID-19 Burnout Scale, the Connor-Davidson Resilience Scale, and the Brief Symptom Inventory were used to investigate 388 college students from Nanjing City, China. All participants were enrolled in university after 2019, and they participated in the survey voluntarily *via* the Internet. Participants were divided into two groups (isolated group vs. non-isolated group) based on whether or not they had been isolated.

**Results:**

(1) Significantly lower scores were found for all factors in the isolated group; (2) COVID-19 burnout significantly negatively predicted resilience and significantly positively predicted psychological distress (anxiety, depression, and somatization symptoms), while resilience significantly negatively predicted psychological distress; and (3) Resilience mediated the relationship between COVID-19 burnout and psychological distress.

**Conclusion:**

Isolation is a risk factor for psychological distress related to COVID-19. Resilience can buffer psychological distress and help improve Chinese college students' wellbeing during the COVID-19 pandemic.

## Introduction

The outbreak of coronavirus disease 2019 (COVID-19) caused unprecedented stress and disruptions in people's daily lives and public health (1). According to the World Health Organization (WHO), as of 16 August 2020, more than 21.3 million people had been diagnosed with COVID-19, including 76,000 deaths (2), with an overall mortality rate of 2.3% in China (3). COVID-19 caused various mental health problems, such as death distress (4), anxiety (5), and burnout (6). In China, nearly 40% of nursing students were found to have some degree of academic burnout during COVID-19 (7), and in the US, 91% of college students reported being worried about the future health of themselves or their families (8). In China, more than a quarter of the population struggled considerably with high stress and anxiety during the pandemic (9). In a meta-analysis, researchers found that the overall prevalence of anxiety and depression symptoms increased significantly after the COVID-19 outbreak in China (10). As mentioned above, COVID-19-related stress has a significant impact on people's physical and mental health, and it has presented great challenges to the health of college students in China. Understanding how COVID-19 leads to psychological distress would aid in developing useful strategies to help young people, such as college students.

As the COVID-19 pandemic continues, the initial shock has gradually taken the form of chronic stress. Thus, burnout is gaining increasing attention. In general, burnout is considered a prolonged response to work-related chronic emotional and interpersonal stress (11). Burnout has been described more as a state of irritability than as a specific clinical condition (e.g., anxiety or depression). Thus, burnout and psychological disorders are seen as distinct concepts (12). Yildirim and Solmaz describe the psychological symptoms caused by prolonged exposure to emotionally demanding and interpersonally stressful situations during the COVID-19 pandemic as “COVID-19 burnout” (6). Some evidence suggests that COVID-19-related stress leads to similar symptoms of burnout as other types of stress outside the workplace. For example, parents suffering from burnout resulting from their children's health problems were found to be more likely to engage in child abuse, indifference, or maltreatment during COVID-19 (13). COVID-19-related burnout problems have also been observed among health professionals (14), nurses (15), and teachers (16). To assess COVID-19 burnout in the general public, the COVID-19 Burnout Scale (COVID-19-BS) was developed (6) based on the Burnout Measure-Short Version (BMS) developed by Malach-Pines (17). The reliability and validity of the COVID-19-BS have been demonstrated in several countries, including Turkey (6), Poland (18), and China (19). As COVID-19 burnout is associated with adverse health outcomes, including anxiety and depression (12), systematically studying it would help us understand the long-term impact of COVID-19 on people, consequently providing a basis for measures to help them.

Resilience is considered a personal characteristic that can help individuals preserve mental health in situations of severe stress or trauma (20). It indicates better coping results in stressful situations, good internal control, better social adaptation, better self-image, and optimism and correlates with positive mental and physical health outcomes (21). A growing body of literature demonstrates that resilience helps individuals in counteracting depression, anxiety, and other negative mental health conditions (22). Resilience can also help alleviate the adverse psychological outcomes associated with COVID-19 (23). Moreover, resilience is also seen as an important influencing factor in burnout. Some evidence suggests that resilience would help counter emotional exhaustion and low professional achievement, which are domains of burnout (24). Resilience was found to be negatively related to burnout symptoms in general (25) and to the emotional exhaustion dimension of burnout among nurses during COVID-19 (26). Another study found resilience to be negatively associated with symptoms of burnout, posttraumatic stress, anxiety, and depression (15). These findings suggest that resilience is a protective factor in the psychological impact of the COVID-19 pandemic on people. With the continued effects of the COVID-19 pandemic, especially in the form of chronic stress, the role and value of resilience deserve more attention, and thus, this study aimed to examine the same.

There is a lack of consensus on the role of resilience in the relationship between burnout and psychological stress. Some researchers have reported that resilience can minimize and buffer the negative mental health consequences of workplace stress (27). Hao emphasized that resilience mediates the impact of burnout on psychological stress (28). Other researchers have reported contrary results. A study on health workers demonstrated that depression has an indirect effect on the dimensions of burnout, which are partially mediated by resilience (29). An overlap between depressive symptoms and burnout has also been suggested (30), and burnout has been considered a precursor of depression (31). Other researchers have emphasized that there are obvious differences between burnout and psychological distress (12). The cognitive profile associated with burnout was found to be significantly different from that associated with depression (32). However, overall, the relationship between burnout and psychological symptoms remains unclear (33).

The situation faced by Chinese college students offers us a window to explore the relationship between burnout and psychological distress. In China, since 2019, college students have been frequently restricted inside their campuses due to the pandemic, greatly affecting their regular lives. The lockdown policy is implemented commonly by local governments in response to the COVID-19 pandemic. This makes Chinese college students entering university after 2019 a special group. On the one hand, most students have experienced lockdown on the campus together with their classmates; on the other hand, some students have been isolated from the campus occasionally due to the pandemic. We posited that being under lockdown together with classmates on campus created a chronic stress condition, which could easily stir up burnout, and being isolated out of campus created an acute stress environment, likely to cause negative mental health symptoms. Thus, the students in this study were divided into two groups (i.e., isolated vs. non-isolated) to facilitate the discussion of the relationship between burnout and psychological distress.

This study explores (1) the incidence of COVID-19 burnout among Chinese students who entered universities after the outbreak of COVID-19 in China; (2) the relationships among resilience, COVID-19 burnout, and mental health factors in these Chinese college students. Thus, the following hypotheses were tested: (H1) there are significant differences between the isolated and non-isolated groups in resilience, burnout, and mental health factors; (H2) resilience mediates the effect of COVID-19 burnout on psychological distress.

## Methods

### Participants

A total of 388 Chinese college students (men = 29.4%; women = 70.6%) participated in this online survey. The grade distribution was as follows: 19.8% in Grade 1, 57.2% in Grade 2, 22.4% in Grade 3, and 0.5% in Grade 4. None of the participants reported having been infected with COVID-19. Eight participants (2.1%) reported that their relatives had been infected with COVID-19 (refer to Table 1). However, 31.2% of the participants (isolated group) reported having been quarantined by the local government or schools owing to the policy for preventing COVID-19. Regarding the family's economic situation, 12.6% reported a below average economic status; 79.6% reported an average economic status; and 7.7% reported an above average economic status.

**Table 1:** Characteristics of the sample (*N* = 388).

**Variable**	**Group**	***n***	**%**	**① *t*/*F*(Sig.)**	**② *t*/*F*(Sig.)**	**③ *t*/*F*(Sig.)**	**④ *t*/*F*(Sig.)**	**⑤ *t*/*F*(Sig.)**	**⑥ *t*/*F*(Sig.)**
Gender	Male	114	29.4	−0.03 (0.97)	1.93 (0.06)	−0.96 (0.34)	−1.23 (0.22)	−1.09 (0.28)	−0.32 (0.75)
	Female	274	70.6						
Grade	One	77	19.8	0.62 (0.60)	0.38 (0.77)	0.39 (0.76)	0.43 (0.74)	0.32 (0.81)	0.36 (0.78)
	Two	222	57.2						
	Three	87	22.4						
	Four	2	0.5						
Family's economic conditions	Below average	49	12.6	0.65 (0.52)	2.63 (0.07)	0.13 (0.88)	0.22 (0.81)	0.09 (0.91)	1.12 (0.33)
	Average	309	79.6						
	Above average	30	7.7						
Has diagnosed relatives	Yes	8	2.1						
	No	380	97.9						
Isolated students	Yes	121	31.2	0.36 (0.72)	0.28 (0.78)	−0.71 (0.48)	−0.56 (0.58)	−0.68 (0.50)	−0.71 (0.48)
	No	267	68.8						

①, COVID-19 burnout; ②, resilience; ③, psychological distress; ④, somatization; ⑤, anxiety; ⑥, depression.

*t*-test is for the bivariate group, *F*-test is for the multivariable group.

Psychological distress factor consists of depression, anxiety, and somatization.

### Measures

#### COVID-19 Burnout Scale

COVID-19 Burnout Scale (6) was used in this study to assess burnout resulting from the COVID-19 pandemic. This questionnaire has been adapted from the Burnout Measure-Short Version (17). It consists of a one-dimensional factor with 10 items, rated on a five-point Likert scale ranging from 1 (never) to 5 (always). The sum of the scores on the 10 items constitutes the total score on the scale, which is used to evaluate individuals' degree of burnout during the pandemic. Cronbach's alpha of this scale was 0.90 in this study.

#### Connor–Davidson Resilience Scale 10-Item Version

The short version of the Connor–Davidson Resilience Scale 10-Item Version (CD-RISC) was used in this study to assess the resilience level of college students (34). This scale consists of 10 items rated on a 5-point Likert scale ranging from 0 (never) to 4 (almost always). CD-RISC-10 has been used widely worldwide, and it has been reported as suitable for Chinese college students (35). In this study, Cronbach's alpha was 0.93.

#### Brief Symptom Inventory-18

The Brief Symptom Inventory-18 (BSI-18) (36) is the short version of the Symptom-Checklist-90 (SCL90). This scale comprises 18 items used to evaluate three types of psychological distress, i.e., anxiety, depression, and somatization. The items are rated on a 5-point Likert scale ranging from 0 (not at all) to 4 (always). In this study, Cronbach's alpha values of the anxiety, depression, and somatization subscales were 0.92, 0.87, and 0.89, respectively. Cronbach's alpha of the total scale was 0.95.

### Procedure

We developed online versions of the questionnaires using the Questionnaire Star Survey (an online data collection platform, https://www.wjx.cn/). Once the participants signed the informed consent form, they were sent the questionnaire *via* the Internet.

The inclusion criteria were being a university student, being of sound health, being aged over 18 years, studying in universities in the past 3 years after the outbreak of the COVID-19 epidemic, and having access to an electronic device. This study was approved by the ethics committee of the Affiliated Hospital of Nanjing University of Traditional Chinese Medicine (Jiangsu Provincial Hospital of Traditional Chinese Medicine).

### Data analysis

SPSS 22.0 and AMOS for Windows (IBM) were used for data analysis. The data were analyzed using *t*-tests and bivariate correlation analysis. PROCESS 3.3 (37) was used to analyze the mediating effects between the variables.

## Results

There was no significant difference found in demographic variables (refer to Table 1). Only the factor of resilience in different economic conditions is close to a significant difference (*F* = 2.63, *p* = 0.073). The score of resilience in the average economic condition group was found significantly lower than that of the above-average group (*p* < 0.05). Other factors, such as COVID-19 burnout and psychological distress (including somatization, anxiety, and depression), were found no difference in demographic variables.

An independent sample *t*-test showed that different isolation conditions (isolated group, *n* = 121; non-isolated group, *n* = 267) showed significant differences in COVID-19 burnout (*t* = 2.42, *p* < 0.05), somatization symptoms (*t* = 2.33, *p* < 0.05), anxiety symptoms (*t* = 2.71, *p* < 0.05), depressive symptoms (*t* = 3.17, *p* < 0.05), and psychological distress (*t* = 2.99, *p* < 0.05), but not in resilience (*t* = −1.37, *p* > 0.05) (refer to Table 2).

**Table 2:** *T*-test for the different factors between isolated and non-isolated participants.

**Variable**	**Isolated (*N* = 121)**	**Non-isolated (*N* = 267)**	***t***	**Sig**.
	***M* ±SD**	***M* ±SD**		
1. COVID-19 burnout	26.16 ± 7.97	24.21 ± 7.05	2.42	0.02
2. Resilience	35.07 ± 6.75	36.04 ± 6.33	−1.37	0.17
3. Psychological distress	1.84 ± 0.57	1.66 ± 0.51	2.99	0.00
Somatization	1.79 ± 0.60	1.64 ± 0.57	2.33	0.02
Anxiety	1.82 ± 0.64	1.64 ± 0.55	2.71	0.01
Depression	1.90 ± 0.61	1.70 ± 0.55	3.17	0.00

From Table 3, it can be seen that all variables were significantly correlated. COVID-19 burnout was found to be significantly negatively correlated with anxiety (*r* = 0.41, *p* < 0.01), depression (*r* = 0.42, *p* < 0.01), somatization (*r* = 0.31, *p* < 0.01), and psychological distress (*r* = 0.41, *p* < 0.01) and positively correlated with resilience (*r* = −0.24, *p* < 0.01). Resilience was significantly negatively correlated with COVID-19 burnout (*r* = −0.208, *p* < 0.01), anxiety (*r* = −0.309, *p* < 0.01), depression (*r* = −0.358, *p* < 0.01), and somatization (*r* = −0.173, *p* < 0.01).

**Table 3:** Descriptive statistics and correlation analysis of the various factors (*N* = 388).

**Variable**	**M**	**SD**	**α**	**1**	**2**	**3**	**Anxiety**	**Depression**
1. COVID-19 burnout	24.82	7.39	0.9					
2. Resilience	35.74	6.47	0.93	−0.208^**^				
3. Psychological distress	1.72	0.53	0.95	0.409^**^	−0.305^**^			
Anxiety	1.7	0.59	0.92	0.405^**^	−0.309^**^	0.950^**^		
Depression	1.76	0.58	0.87	0.415^**^	−0.358^**^	0.910^**^	0.836^**^	
Somatization	1.69	0.59	0.89	0.305^**^	−0.173^**^	0.890^**^	0.778^**^	0.668^**^

^*^p < 0.05.

** *p* < 0.01.

**Figure 1:**
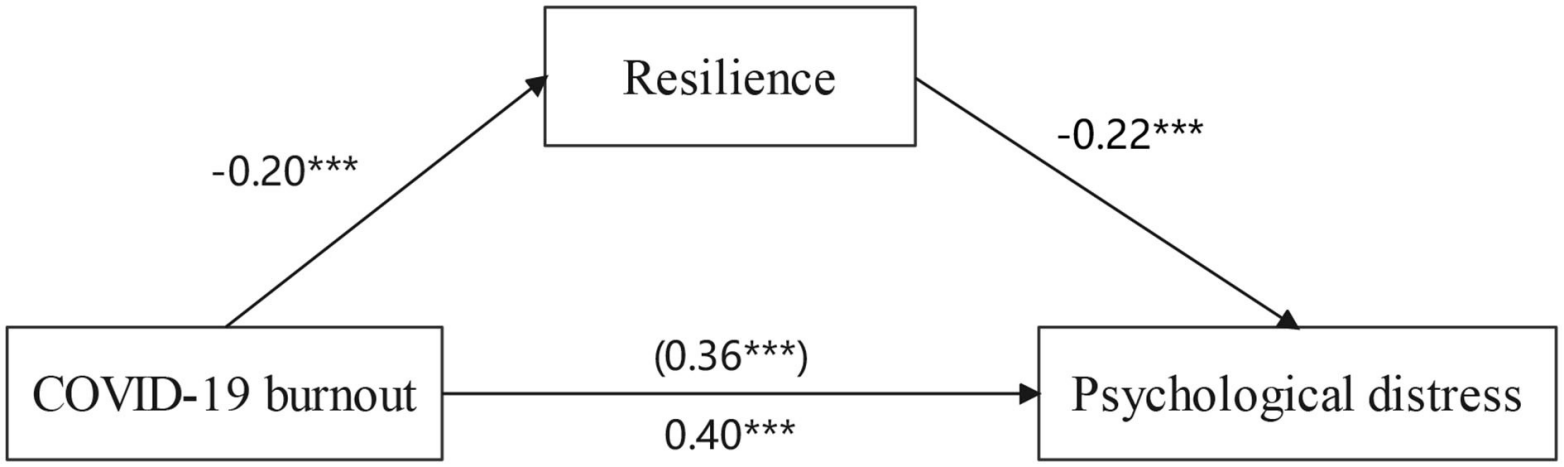
Mediation effect of resilience between COVID-19 burnout and psychological distress. ***p < 0.001.

Based on the correlation analysis, the mediating effect of resilience in the relationship between COVID-19 burnout and psychological distress (anxiety, depression, and somatization) was analyzed beyond demographic variables (refer to Figure 1 and Table 4). COVID-19 burnout significantly predicted resilience (β = −0.20, *p* < 0.001) and explained 7% of the variance in resilience. The direct effect of COVID-19 burnout on psychological distress (β = 0.40, *p* < 0.001) was significant, 0.026, 95% CI (0.019, 0.032). The value of the indirect effect was 0.003, 95% CI (0.001, 0.007), indicating that COVID-19 burnout significantly predicted psychological distress (β = 0.36, *p* < 0.001) through resilience (β = −0.22, *p* < 0.001). Table 5 presents the indirect effect estimates in unstandardized coefficients.

**Table 4:** Mediating effect analysis of the hypothesized mediation model.

**Path: COVID-19 burnout → resilience → psychological distress**	**Effect value**	**Boot SE**	**Boot CL lower**	**Boot CL upper**	**Relative effect value**
**Total effect**	0.029	0.003	0.022	0.036	
**Direct effect**	0.026	0.003	0.019	0.032	88.93%
**Indirect effect**	0.003	0.002	0.001	0.007	11.07%

**Table 5:** Unstandardized coefficients for the hypothesized mediation model.

**Antecedent**	***M*** **(resilience)**						***Y*** **(psychological distress)**					
	**Coeff**	**SE**	***t***	***p*-Values**	**95%**		**Coeff**	**SE**	***t***	***p*-Values**	**95%**	
					**LLCI**	**ULCI**					**LLCI**	**ULCI**
***X*** **(COVID-19 burnout)**	−0.18	0.04	−4.04	0.00	−0.26	−0.09	0.03	0.00	7.66	0.00	0.02	0.03
***M***	–	–	–	–	–	–	−0.02	0.00	−4.72	0.00	−0.03	−0.01
**Constant**	39.60	5.74	6.90	0.00	28.31	50.90	1.41	0.46	3.10	0.00	0.52	2.31
	*R*^2^ = 0.07						*R*^2^ = 0.24					
	*F* = 3.47, *p* < 0.001						*F* = 12.97, *p* < 0.001					

SE, standard error; Coeff, unstandardized coefficient; X, independent variable; M, mediator variable; Y, outcome variable.

## Discussion

The results of this study indicate that there were significant differences between the isolated and non-isolated groups. As seen from Table 2, the isolated group scored significantly higher on depression, anxiety, and somatization than the non-isolated group, even though none of the participants reported ever being diagnosed with COVID-19.

First, consistent with previous studies, our data highlight the negative influence of COVID-19 on the mental health of students who had experienced isolation. One study revealed that 91% of the participating students worried about the health of their own and their families during the COVID-19 pandemic (8). COVID-19-related risk factors have been reported to cause various mental health problems, such as anxiety and affective disorders (5). Therefore, paying attention to the continued impact of the COVID-19 pandemic is crucial.

Second, our data support that isolation, and not merely lockdown, may have a negative impact on the mental health of college students. Since the outbreak of COVID-19, isolation and social distancing have been the main measures of protection against the risk of COVID-19 infection worldwide (38). Some evidence shows that a “home quarantine” lockdown increased psychological distress in college students during severe acute respiratory syndrome (SARS) in 2001 (39). The COVID-19 pandemic seems to have created a similar situation. In China, more than 25% of the population experienced high levels of stress and anxiety during the COVID-19 pandemic (9). However, a meta-analysis concluded that COVID-19 lockdowns did not impact mental health (40). A longitudinal study on the mental health status of Chinese college students also found no difference before and after being isolated on campus due to the COVID-19 pandemic (41). One possible reason for the discrepancies in the results of various studies may be that isolation and lockdown were not clearly defined. Our results show that students in the isolated group demonstrated higher mental health risks than those in the non-isolated group (being under lockdown with classmates on campus). In other words, at least in the case of Chinese college students, isolation from peers may be an important factor affecting individual mental health during the COVID-19 pandemic.

In this study, significant differences were found in the psychological distress factors between the isolated and non-isolated groups, but no significant difference was detected in other demographic variables. Additionally, no difference was found in resilience scores based on differences in the demographic variables. This seems to indicate that pandemic-related burnout is the premise of various psychological symptoms. Thus, at least among the participants of our study, burnout occurred first, followed by psychological symptoms. Repeated negative work experiences and accumulation have previously been shown to exacerbate burnout into depressive symptoms (42). Our results may imply that COVID-19-related burnout would accumulate over time and eventually develop into symptoms of psychological distress. This finding also supports the idea that burnout is a disparate construct from depression and anxiety (12).

In our study, higher COVID-19-related burnout is correlated with higher anxiety, depression, and somatization (refer to Table 2). Studies have reported that COVID-19 burnout is related to many negative consequences on mental health (18). In Spain, 40.1% of internal medicine physicians suffered from burnout syndrome during the COVID-19 outbreak (43). Our data indicate a trend similar to this finding, suggesting that more attention should be paid to COVID-19-related burnout among isolated college students in the future.

As for the relationships among burnout, resilience, and psychological distress, the present data suggest that COVID-19 burnout affects psychological distress through resilience. Some researchers have found that resilience minimizes and buffers the negative influence of stress on mental health and plays a mediating role in the relationship between burnout factors and mental health symptoms (27). However, this finding is contrary to Serrao's study, which reported that depression affects burnout through resilience (29). Koutsimani et al. (12) suggested that burnout and psychological symptoms overlap in normal circumstances and are difficult to distinguish. As mentioned earlier, resilience is a personal protective characteristic, considered to be a process of recovery from trauma or severe stress (44), and a factor that can help in improving psychological health (6).

The present findings provide us with an overall picture of the psychological impact of the COVID-19 pandemic in China. According to the conservation of resources (COR) theory (45), individuals with more resources are more likely to cope better and survive in the face of adversities. When individuals face severe stress, their resources are slowly consumed in the process of coping with the stressor; once resources are consumed to a certain degree, as a signal of resource depletion, various symptoms appear. In the early stage of COVID-19, most people were shocked by the unprecedented situation, and various psychological symptoms appeared as a result of COVID-19-related stress. However, with the protective effect of resilience, psychological symptoms were assuaged, and people recovered slowly to a new fragile balance. This may be why some reports have found that COVID-19 lockdowns did not influence mental health (40) and that there was no difference between Chinese students before and after being isolated on campus (41). Of particular interest is the fact that when the lockdown was implemented again, various psychological symptoms recurred, which is reflected in the results of this study. Therefore, the overlap between burnout and psychological symptoms (12) may occur at the beginning of stress, and the separation of burnout and psychological symptoms may occur more in the process of stress relief. Thus, it may be necessary to explore the relationships among burnout, resilience, and psychological symptoms in different environments.

This study has some limitations. First, Chinese college students were the research participants; thus, the results need to be interpreted cautiously when being extended to other groups. Second, the questionnaire survey design limits the in-depth exploration of the participants' emotional states. Therefore, the results of this study should be further verified through qualitative interviews in the future.

## Data availability statement

The original contributions presented in the study are included in the article/supplementary material, further inquiries can be directed to the corresponding author.

## Ethics statement

The studies involving human participants were reviewed and approved by the Ethics Committee of The Affiliated Hospital of Nanjing University of Traditional Chinese Medicine (No. 2022NL-134-02). The patients/participants provided their written informed consent to participate in this study.

## Author contributions

Conception, design, and provision of study materials or patients: YS. Administrative support: YS, XZ, and ZZ. Collection and assembly of data: YS, SZ, and GC. Data analysis and interpretation: YS, SZ, and SY. Manuscript writing and final approval of manuscript: All authors. All authors contributed to the article and approved the submitted version.

## Funding

This study was supported by the Major Research Project of Jiangsu University Philosophy and Social Science (2020SJZDA125) to YS, the Science and Technology Project of Jiangsu Bureau of Traditional Chinese Medicine (YB201906) to XZ, and the Jiangsu Educational Science 14th Five-Year-Plan Project (B/2021/01/34) to ZZ.

## Conflict of interest

The authors declare that the research was conducted in the absence of any commercial or financial relationships that could be construed as a potential conflict of interest.

## Publisher's note

All claims expressed in this article are solely those of the authors and do not necessarily represent those of their affiliated organizations, or those of the publisher, the editors and the reviewers. Any product that may be evaluated in this article, or claim that may be made by its manufacturer, is not guaranteed or endorsed by the publisher.
